# Inhibitory Effects of Bioassay-Guided Isolation of Anti-Glycation Components from *Taraxacum coreanum* and Simultaneous Quantification

**DOI:** 10.3390/molecules23092148

**Published:** 2018-08-27

**Authors:** Kang Hee Lee, Wan Kyunn Whang

**Affiliations:** Pharmaceutical Botany Laboratory, College of Pharmacy, Chung-Ang University, Heukseok-dong, Dongjak-gu, Seoul 156-756, Korea; 2_kanghee@naver.com

**Keywords:** *Taraxacum coreanum*, bioassay-guided isolation, type-2 diabetes, diabetic complication, AGE formation, MGO, glucosidase, Simultaneous analysis, flavones, hydroxycinnamic acid

## Abstract

Inhibition of the formation of advanced glycation end products (AGEs) is an attractive strategy in diabetes treatment. *Taraxacum coreanum* extracts were suggested to have antidiabetic effects. However, studies on the components of *T. coreanum* are lacking, and there is no report on the inhibitory effects of *T. coreanum* on the formation of AGEs. Therefore, *T. coreanum* extracts and fractions were tested for their inhibitory effects on α-glucosidase and AGEs formation in two systems (bovine serum albumin (BSA)–glucose and BSA–methylglyoxal (MGO)). Bioassay-guided isolation of compounds from *T. coreanum* led to six flavones (**1**–**6**) and four hydroxycinnamic acid derivatives (**7**–**11**). Compound **11** exhibited the highest inhibitory activity against α-glucosidase and AGEs formation and had the highest content in *T. coreanum* extract. All compounds except compound **9** showed a stronger inhibition than the positive control in the BSA-glucose and BSA-MGO system. In addition, *T. coreanum* showed a higher content of bioactive compounds and stronger inhibition of AGE formation and α-glucosidase activity than *T. officinale*. Our study demonstrated the preventive and therapeutic efficacy of *T. coreanum* and its potential use as a cost-effective phytopharmaceutical in complementary therapy against type-2 diabetes and its complications.

## 1. Introduction

Protein glycation, also called Maillard reaction, is a nonenzymatic reaction that occurs between the amino and carbonyl groups of proteins, lipids, and nucleic acids and results in the formation of a group of heterogeneous compounds called advanced glycation end products (AGEs). These compounds form covalent bonds with proteins, which results in changes in the structural and functional properties of proteins. The interaction between AGEs and their receptors (RAGE) causes oxidative stress, thrombosis, and inflammatory reactions [1,2]. The rate of AGE formation is accelerated in diabetes [3]. Unless long-term uncontrolled, it can lead to a series of complications such as cataracts, atherosclerosis, neuropathy, retinopathy, nephropathy, and delayed wound healing. The tissue concentrations of AGE were twofold higher in diabetic patients with end-stage renal disease than in diabetic patients without renal disease [4]. Additionally, diabetic patients with Alzheimer’s disease had increased accumulation of AGEs and upregulated RAGE in the brains [5], and patients with heart failure-associated cardiac stiffness showed myocardial accumulation of AGEs [6]. AGEs formation can be suppressed through use of inhibitors [7]. Some synthetic compounds, such as aminoguanidine (AMG) exhibit a high AGE-inhibitory activity; however, they are associated with various adverse effects in vivo and are not suitable for food applications [7]. Thus, research on natural products that can inhibit the formation of AGEs has recently increased [8]. Antioxidants, which include plant extracts and their active ingredients, are widely used to inhibit the formation of AGEs in vivo and in food [9]. Amadori products, such as deoxyglucosones, glyoxal, and methylglyoxal (MGO), are intermediates produced during AGEs formation in humans [10].

Dandelions (Taraxacum) is a member of the Asteraceae family and grows widely on roadsides and rural sites located in warm temperate zones. There are 30–57 varieties of Taraxacum, and many microspecies are divided into nine sections. Taraxacum species have been used as a herbal medicine for a long time [11,12]. *Taraxacum coreanum* NAKAI, known as “white dandelion”, is native to Korea and Japan and grows mainly in South Korea. Moreover, *T. coreanum* has been reported to have various biological activities, including antioxidant [13], anti-inflammatory [14], anti-fungal [15], and anti-cancer [16]. In addition, polysaccharides, b-sitosterol, daucosterol, taraxasteryl acetate, chrysoeriol, diosmetion, luteolin, luteolin-7-glucoside, esculetin, 5-hydroxypyttolidin-2-one, taraxinic acid, and taraxinic acid-1′-β-d-glucopyranoside were isolated from *T. coreanum* [16,17]. According to previous studies [18,19], *T. coreanum* had a higher content of phenolic compounds, antioxidant activity, and tyrosinase inhibitory activity than *T. officinale*, which was commonly known as dandelion. Additionally, *T. coreanum* extracts were reported to show anti-diabetes effects [20]. However, studies on the components of *T. coreanum* are still lacking, and there is no report on the inhibitory effects on the formation of AGEs of *T. coreanum* as well as *T. offcinale*. Therefore, this study was carried out to isolate the major components from *T. coreanum* extracts and evaluate their inhibitory effects on α-glucosidase activity, which is related to diabetes, and the formation of fluorescent AGEs in bovine serum albumin (BSA)/glucose and BSA/methylglyoxal (MGO) systems using fluorescence spectroscopy. In addition, we evaluate the excellence of *T. coreanum* compared to *T. offcinale*.

## 2. Results and Discussion

### 2.1. AGE Formation in BSA-glucose and BSA-MGO Systems, and α-Glucosidase Inhibitory Activities of the Extracts and Fractions from T. coreanum

Accumulation of AGEs in the body is implicated in the development of chronic degenerative diseases [21]. AGE inhibition is a therapeutic option for diabetes that is not based on controlling of postprandial blood glucose level. This approach could be useful in the prevention or reduction of diabetic complications [22]. Therefore, studies have been performed to develop AGE inhibitors. Owing to the importance of AGEs in the pathogenesis of various related diseases, AGE inhibitors have received increased attention. methylglyoxal (MGO) is a crucial intermediate for the formation of AGEs in vivo [10]. In addition, α-glucosidase inhibitors are believed to act as anti-diabetic agents by impeding sugar degradation and attenuating postprandial hyperglycemia. Thus, inhibition of α-glucosidase activity and carbohydrate hydrolysis would be beneficial for controlling blood glucose levels in diabetic patients [23]. In this study, inhibitory effects on AGE formation were determined in bovine serum albumin (BSA)-glucose and BSA-MGO systems to demonstrate the effects of *T. coreanum* in preventing diabetic complications. We also examined α-glucosidase inhibitory activity of *T. coreanum* to demonstrate its potential in preventing diabetes. The results are summarized in Table 1. 

The extract with 100% methanol (Ext), n-Hexane (Hx), dichloromethane (DCM), and EA fractions from *T. coreanum* exhibited inhibition of α-glucosidase (IC_50_ values of 1623.08 ± 184.40, 1270.70 ± 72.58, 2041.44 ± 469.46, and 544.53 ± 36.46 μg/mL, respectively). Similar to the results of Kim et al. [24], *T. coreanum* extracts showed stronger inhibition (IC_50_ values of 444.97 ± 55.86 µg/mL) than *T. officinale* extracts (IC_50_ values of 2195.89 ± 267.35 µg/mL). Regarding the inhibitory activity of AGE formation in both BSA-glucose and BSA-MGO systems, EA fractions of *T. coreanum* (IC_50_ values of 119.47 ± 12.06 and 127.47 ± 23.87 µg/mL) exhibited the highest inhibition, while the Hx fraction had mild activity. Among *T. coreanum* and *T. officinale* extracts, *T. coreanum* showed higher inhibition in both BSA-glucose and BSA-MGO systems (IC_50_ values of 183.97 ± 28.52 and 98.82 ± 2.51) than *T. officinale* (IC_50_ values of 201.25 ± 33.99 and 105.37 ± 24.42 µg/mL).

### 2.2. Identification of Compounds ***1***–***11*** Isolated from T. coreanum

**Figure 1:**
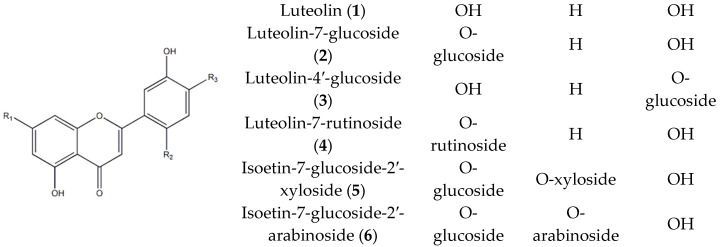

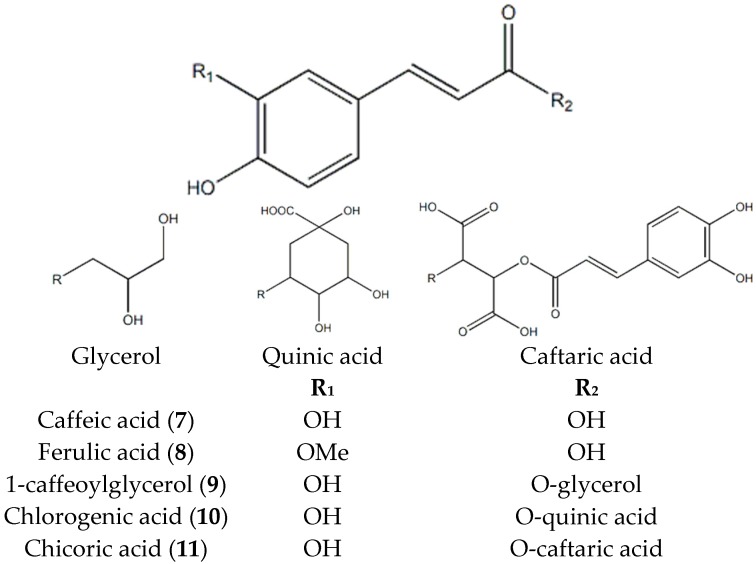
Structures of the compounds **1**–**11**.

**Figure 2:**
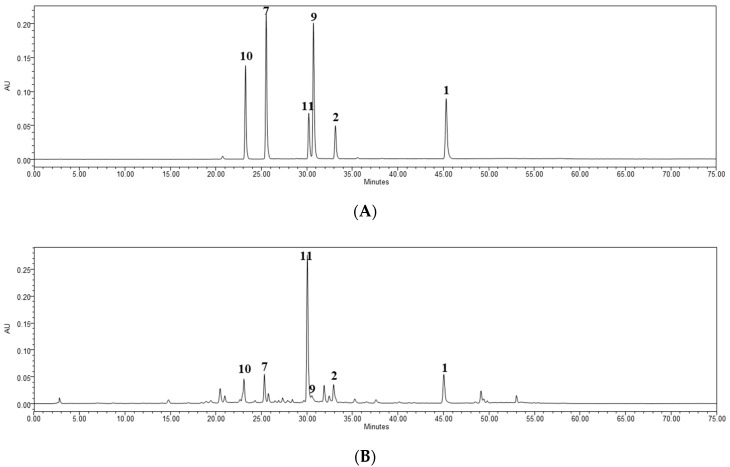
Chromatograms of (**A**) standards mixture and (**B**) *T. coreanum* ext.

Chromatographic separation of ethyl acetate (EA), n-butanol (BuOH), and water fractions from *T. coreanum* were performed by bioassay-guided isolation. The results revealed that six flavones (**1**–**6**) and four hydroxycinnamic acids (**7**–**11**) were isolated from EA, BuOH, and water fractions. Compounds **1**–**11** isolated from *T. coreanum* were identified as luteolin (**1**) [25], luteolin-7-glucoside (**2**) [25], luteolin-4′-glucoside (**3**) [26], luteolin-7-rutinoside (**4**) [27], isoetin-7-glucoside-2′-xyloside (**5**) [28,29], isoetin-7-glucoside-2′-arabinoside (**6**) [28,29], caffeic acid (**7**) [30,31], 1-caffeoylglycerol (**8**) [32], ferulic acid (**9**) [33], chlorogenic acid (**10**) [34], and chicoric acid (**11**) [35] by comparing spectroscopic (^1^H-, ^13^C-NMR) and LC-MS data from previous studies (Figure 1). The *m*/*z* data and the retention time of each compound are shown in Table 2. The observed mass value accuracy of compounds **1**–**11** is within the 5 ppm range, indicating that the results are credible. HPLC analysis was performed to determine the major components of *T. coreanum* extracts. (Figure 2). The inhibitory activity of compounds **1**–**11** on α-glucosidase and AGE formation was also determined (Table 3).

### 2.3. AGE Formation in BSA-glucose and BSA-MGO Systems and α-Glucosidase Inhibitory Activities of Compound ***1***–***11*** Isolated from T. coreanum

Regarding AGE formation inhibitory activity in BSA-glucose system, glycosylation at the C-7 position of flavones improved chelation effect and glycosylation at the C-4′ position of flavones decreased the inhibitory capacity [36]. It was same result that the lower inhibitory activity of compound **3**, which was glycosylated at C-4′, had than that of compounds **2**–**6** (glycosylation at the C-7 position). In addition, compound **2**, which was glycosylated at the C-7 position of luteolin-type flavonols, showed the highest inhibitory activity, followed by luteolin (compound **1**) with no glycosylation, and compounds **5** and **6** with glycosylation at the C-7 and C-2′ position of isoetin-type flavonols. The IC_50_ values of compounds **2**–**6** were 122.81 ± 2.02, 423.30 ± 18.04, 253.3 ± 18.04, 268.18 ± 3.41, and 238.05 ± 13.82 μM, respectively. In contrast, compound **3** (only glycosylation at C-2′ position) had the lowest IC_50_ values with 423.30 ± 18.04 mM. Among hydroxycinnamic acids, derivatives of both caffeic acid and tartaric acid (compound **11**) showed higher inhibitory activity than derivatives of caffeic acid and quinic acid (compound **10**). Compounds **8** and **9** showed mild activity, with IC_50_ values of 324.21 ± 8.29 and 306.99 ± 10.16 μM. Compound **10** demonstrated slight inhibition, with IC_50_ value of 704.86 ± 167.44 μM.

In BSA-MGO system, compound **1** showed the highest inhibitory activity with IC_50_ value of 66.11 ± 17.06 μM, followed by compounds **2** and **3,** which was glycosylated at the C-7 or C-4′ position of flavones, and compounds **4**–**6**, which had two glycosylations. The inhibitory capacity of compounds **4**–**6** was weaker than that of other flavone compounds, but stronger than compounds **8**–**9**. Among hydroxycinnamic acids, compounds **9**–**11**, which have a glycerol group, quinic acid, or caftaric acid, showed similar inhibitory potentials, with IC_50_ values of 140.72 ± 67.36, 138.18 ± 1.91 μM, and 141.21 ± 8.76, respectively. Compound **8**, which had an IC_50_ value of 151.67 ± 65.36, exhibited weaker inhibitory activities than flavones and other compounds.

Finally, regarding α-glucosidase activity, compounds **3**, **4**, and **11** possessed the most potent activity (IC_50_ value of 598.24 ± 146.52, 670.50 ± 50.83, and 639.25 ± 12.51 μg/mL, respectively). Compounds **10** and **2** were the second most potent α-glucosidase inhibitors (IC_50_ value with 1148.67 ± 162.05 and 1455.95 ± 162.32 μg/mL, respectively), followed by compounds **7** and **8** (IC_50_ value of 5134.55 ± 803.54 and 2951.13 ± 3.94 μg/mL, respectively). In contrast, compounds **1**, **5**, **6**, and **9** demonstrated no inhibitory activity and had IC_50_ values >500 μM or were ND. The results are summarized in Table 2.

### 2.4. Quantitative HPLC Analysis of Six Bioactive Compounds

**Figure 3:**
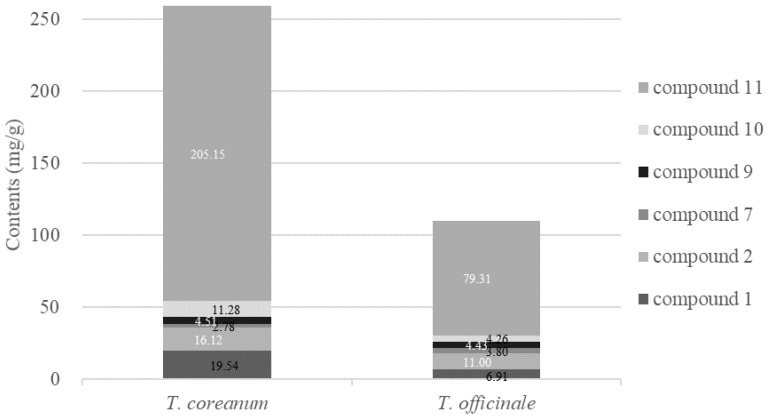
Content of major compounds in *Taraxacum coreanum* and *T. officinale.*

HPLC analysis was performed for quantitative evaluation of the active components of *T. coreanum* extract (Figure 2). After a preliminary screening of the collected samples, compound **11** was identified as the major component of *T. coreanum* extract, and compounds **9** and **10** were the second major components. The six compounds (**1**, **2**, **7**, **9**–**11**) that showed the most potent inhibitory activity against AGE formation were examined. To establish a quality-control standard, this study developed a standard extraction method. The six major compounds were extracted using different solvent compositions and extraction times (Table 4). These six compounds were extracted with 50% ethanol (216.69 mg/g), and the content was extracted from 30 to 90 min stably. Based on the contents of the six compounds, 30–90 min extraction with 50% ethanol was the optimized solvent condition. Among *T. coreanum* and *T. officinale,* the contents of all compounds except compound **7** were higher in *T. coreanum* than in *T. officinale*, but the content of compound **7** was significantly lower than that of other compounds. In particular, the content of compound **11**, which is the major bioactive compound, is about more 125.84 (mg/g) than *T. officinale*. (Figure 3). The quantitative study suggested that the IC_50_ values of the tested samples were inversely proportional to the total content of compounds **1**, **2**, **7**, **9**–**11**, indicating that these six compounds could play important roles in the inhibition of AGE formation. This finding suggested that, for medicinal purposes, HPLC analysis of these six compounds cac be performed for obtaining quality-control standards of *T. coreanum*. Actually, the *T. coreanum* extraction (extracted with 50% ethanol) demonstrated better inhibition activity than the 100% methanol extraction in all assay. In addition, we collected eight samples of *T. coreanum* from different regions, including Gyeongsangnam-do, Gyeongsangbuk-do, Gyeonggi-do, Chungcheongnam-do, Jeollabuk-do, and Gangwon-do. The contents of compounds **1** and **11** were the highest in samples obtained from Gyeongsangnam-do, Sancheong; compound **2** was highest in samples from Gyeongsangbuk-do, Yeongcheon; compound **8** was highest in samples from Gyeongsangnam-do, Sancheong, and Chungcheongnam-do, Taean; and compound **10** was highest in samples from Gyeongsangbuk-do, Yeongcheon and Gangwon-do, Yanggu. The content of compound **9** was 5 mg/g in average, and contained almost the same amount in all samples. Considering the total content of the six major bioactive compounds of *T. coreanum*, the most abundant compounds were harvested from Gyeongsangnam-do, Sancheong. The average content of major components in the extract was calculated based on quantitative analysis. We suggested that *T. coreanum* harvested from Gyeongsangnam-do, Sancheong was the best useful natural alternative medicine for diabetic complications. The results are summarized in Table 5.

## 3. Materials and Methods

### 3.1. Plant Materials

The aerial parts of *T. coreanum* (harvested Jiri-mountain, Sancheong-gun, Korea) were purchased from Kyung-Dong market, Seoul, Korea. In addition, the aerial parts of *T. coreanum* were collected from Sancheong, Yeongcheon, Gimpo, Taean, Jeongeup, Yanggu, and Bonghwa in Korea for analysis. Prof. Whang Wan Kyunn identified *T. coreanum,* which was purchased from Kyung-Dong market. 

### 3.2. Instruments and Reagents

Methanol (MeOH), ethanol (EtOH), Hx, DCM, EA, BuOH, and distilled water were used for extraction, fractionation, and open column chromatography. Sephadex LH-20 (25–100 μm; Pharmacia, Stockolm, Sweden), MCI CHP 20P (Supelco, St. Louis, MO, USA), and ODS gel (400–500 mesh; Waters, Milford, MA, USA) were used for open column chromatography. Dimethyl sulfoxide-*d*_6_ (DMSO-*d*_6_) and methanol-*d*_4_ (CD_3_OD) were used as solvents for NMR analysis. Molecular weight was determined by using ultrahigh performance liquid chromatography and high-resolution mass spectrometry (UHPLC-HRMS) coupled with electrospray ionization hybrid linear trap-quadruple-Orbitrap MS (ESI/LTQ-Orbitrap) using an Ultimate 3000 RSLC (Thermo, Darmstadt, Germany). ^1^H-nuclear magnetic resonance (NMR) spectra were recorded at 600 MHz and ^13^C-NMR spectra were recorded at 150 MHz on a JEOL spectrometer. The chemical shifts are expressed in parts per million (ppm) on the δ scale and as coupling constants (*J*) in Hertz. HPLC was conducted using Empower Pro 2.0 software and determination was performed using WATERS 2695 system pump with a Waters 996 Photodiode array detector (USA). A Waters Kromasil C18 (4.6 × 250 mm, 5 μm) column was used as the separation column. HPLC-grade solvents, such as MeOH and distilled water (H_2_O), were purchased from J. T. Baker^®^ (Phillipsburg, PA, USA). HPLC-grade phosphoric acid was obtained from DEAJUNG Chemical (Siheung, Gyeonggi, Korea). Reagents and solvents, including BSA, d-(−)-fructose, d-(+)-glucose, AMG, MGO, sodium azide, sodium phosphate buffer, α-glucosidase from *Saccharomyces cerevisiae* (Saccharomycetaceae), 4-nitrophenyl α-d-glucopyranoside (p-NPG), potassium phosphate buffer, acarbose, and sodium carbonate were purchased from Sigma-Aldrich Chemical Company (St. Louis, MO, USA). 

### 3.3. Extraction, Fractionation, and Isolation from T. coreanum

The aerial parts of *T. coreanum* (6.5 kg) were dried and powdered prior to extraction with MeOH (20 L × 3) at 20–25 °C. Then, the filtrate was evaporated to dryness (1.17 kg) under reduced pressure, suspended in water (H_2_O), and then partitioned using Hx, EA, DCM, and BuOH. The result yielded Hx (16.6 g), DCM (80.48 g), EA (24.55 g), BuOH (101.93 g), and water (250.34 g) fractions.

The EA fraction was chromatographed with a Sephadex LH-20 solvent system to increase the elution of MeOH:Water (30:70 to 100:0), and it yielded eight sub-fractions. Compound **1** was isolated from sub-fraction 13 using MCI gel chromatography with 70% MeOH. Compound **2** was isolated from sub-fraction 9 using MCI gel chromatography with 50% MeOH. Sub-fraction 10 was separated using Sephadex LH-20 with 50% MeOH to obtain fractions 10-1 to 10-7. Separation of sub-fraction 10-3 using MCI gel chromatography with a 40% MeOH solvent system yielded seven fractions. Sub-fractions 10-3-7 were separated using MCI gel with a 50% MeOH solvent system to collect the fractions. Compound **3** was isolated from fractions 10-3-7-5 using Sephadex LH-20 with 40% MeOH. Sub-fraction 7 was separated using MCI gel column chromatography with 30% MeOH to obtain fractions 7-1 to 7-10. Separation of sub-fraction 7-5 using MCI gel column chromatography with 40% MeOH solvent system yielded four fractions. Compound **4** was isolated from sub-fractions 7-5-1 using Sephadex LH-20 with 40% MeOH. In addition, sub-fraction 7-8 was subjected to MCI gel chromatography with 50% MeOH and sub-fraction 7-8-2 was subjected to ODS column chromatography (50% MeOH) to yield compound **8**. Sub-fraction 4 was separated using Sephadex LH-20 column chromatography with 20% MeOH to obtain fractions 4-1 to 4-5. Separation of sub-fraction 4-2 using MCI gel chromatography with 30% MeOH solvent system yielded four fractions, and compound **7** was isolated from sub-fraction 4-2-1 using ODS column chromatography with an eluent of 50% MeOH. In addition, sub-fraction 4-2-2 was subjected to ODS column chromatography with 50% MeOH to yield compound **9**.

A portion of the BuOH fraction was separated using a Sephadex LH-20 column chromatography with a gradient elution solvent system of 30% to 100% MeOH to give six sub-fractions. Sub-fraction 2 was applied to MCI gel chromatography using a 10% to 60% MeOH gradient elution solvent system to yield sub-fractions 2-1 to 2-12. Compound **5** was isolated from sub-fraction 2-9 using a Sephadex LH-20 with 20% MeOH. In addition, compound **6** was isolated from sub-fraction 2-10 using MCI column chromatography with 50% MeOH. Sub-fractions 2-11 was isolated by recrystallization to yield compounds **10**.

A portion of the water fraction was separated using Diaion HP-20 column chromatography with water, and then separated with 30%, 50%, 80%, and 100% MeOH. Sub-fraction 2 (50% MeOH) was applied to Sephadex LH-20 column chromatography using a 5% MeOH solvent system to yield sub-fractions, and sub-fraction 2-4 was subjected to MCI column chromatograph with 10% MeOH to yield sub-fraction 2-4-2. Sub-fraction 2-4-2 was chromatographed on ODS column with 10% MeOH to yield compound **11**.

### 3.4. Identification of Compounds Isolated from T. coreanum

*Luteolin* (**1**): C_15_H_10_O_6_ ESI/LTQ-Orbitrap-HRMS *m*/*z*: 287.0546 [M + H]^+^; ^1^H-NMR (600 MHz, DMSO-*d*_6_): 7.41 (1H, dd, *J* = 1.8, 8.4 Hz, H-6′), 7.39 (1H, d, *J* = 2.4 Hz, H-2′), 6.89 (1H, d, *J* = 8.4 Hz, H-3′), 6.66 (1H, s, H-3), 6.43 (1H, d, *J* = 1.8 Hz, H-8), 6.18 (1H, d, *J* = 1.8 Hz, H-6); ^13^C-NMR (150 MHz, DMSO-*d*_6_): 181.8 (C-4), 164.3 (C-7), 164.0 (C-2), 161.6 (C-5, 2), 157.4 (C-9), 149.8 (C-4′), 145.9 (C-3′), 121.7 (C-6′), 119.1 (C-1′), 116.15 (C-5′), 113.5 (C-2′), 103.8 (C-10), 103.0 (C-3), 99.0 (C-6), 94.0 (C-8).

*Luteolin-7-glucoside* (**2**): C_21_H_20_O_11_; ESI/LTQ-Orbitrap-HRMS *m*/*z*: 449.1076 [M + H]^+^; ^1^H-NMR (600 MHz, DMSO-*d*_6_): 7.44 (2H, dd, *J* = 7.8, 9.0 Hz, H-2′, 6′), 6.90 (1H, d, *J* = 8.4 Hz, H-3′), 6.78 (1H, s, H-3), 6.74 (1H, s, H-8), 6.43 (1H, s, H-6), 5.07 (1H, d, *J* = 7.8 Hz, H-1′′); ^13^C-NMR (150 MHz, DMSO-*d*_6_): 181.7 (C-4), 164.3 (C-7), 162.7 (C-2), 160.9 (C-5), 156.7 (C-9), 149.8 (C-4′), 145.6 (C-3′), 121.0 (C-6′), 118.9 (C-1′), 115.8 (C-5′), 113.3 (C-2′), 105.1 (C-10), 102.9 (C-3), 99.6 (C-1′′), 99.3 (C-6), 94.5 (C-8), 76.9 (C-5′′), 76.2 (C-3′′), 72.9 (C-2′′), 69.3 (C-4′′), 60.4 (C-6′′).

*Luteolin-4′-glucoside* (**3**): C21H20O11; ESI/LTQ-Orbitrap-HRMS *m*/*z*: 449.1076 [M + H]^+^; ^1^H-NMR (600 MHz, DMSO-*d*_6_): 7.76 (1H, S, H-6′), 7.63 (1H, d, *J* = 9.0 Hz, H-2′), 6.94 (1H, d, *J* = 7.2 Hz, H-3′), 6.75 (1H, s, H-3), 6.50 (1H, s, H-8), 6.16 (1H, s, H-6), 4.86 (1H, d, *J* = 6.6 Hz, H-1′′); ^13^C-NMR (150 MHz, DMSO-*d*_6_): 181.7 (C-4), 164.3 (C-2), 164.0 (C-7), 161.6 (C-52), 157.4 (C-9), 149.8 (C-4′), 145.9 (C-3′), 121.7 (C-6′), 119.1 (C-1′), 116.15 (C-5′), 113.5 (C-2′), 103.8 (C-10), 103.0 (C-3), 102.2 (C-1′′), 99.2 (C-6), 94.3 (C-8), 77.5 (C-5′′), 76.1 (C-3′′), 73.5 (C-2′′), 70.1 (C-4′′), 61.0 (C-6′′).

*Luteolin-7-rutinoside* (**4**): C_27_H_30_O_15_; ESI/LTQ-Orbitrap-HRMS *m*/*z*: 595.1650 [M + H]^+^; ^1^H-NMR (600 MHz, DMSO-*d*_6_): 7.41 (2H, dd, *J* = 9.0, 12.0 Hz, H-2′, 6′), 6.88 (1H, d, *J* = 7.8 Hz, H-3′), 6.70 (1H, s, H-3), 6.66 (1H, s, H-8), 6.41 (1H, s, H-6), 5.04 (1H, d, *J* = 7.8 Hz, H-1′′), 4.5 (1H, s, H-1′′′); ^13^C-NMR (150 MHz, DMSO-*d*_6_): 182.4 (C-4), 165.1 (C-2), 163.4 (C-7), 161.8 (C-5), 157.4 (C-9), 150.7 (C-4′), 146.4 (C-3′), 121.7 (C-6′), 119.8 (C-1′), 116.6 (C-5′), 114.0 (C-2′), 105.9 (C-10), 103.6 (C-3), 101.1 (C-1′′), 100.4 (C-1′′′), 100.0 (C-6), 95.3 (C-8), 76.8, 76.1, 73.6, 72.6, 71.3, 70.8, 70.1, 68.9, 66.6, 18.3.

*Isoetin-7-glucoside-2′-xyloside* (**5**): C_26_H_28_O_16_; ESI/LTQ-Orbitrap-HRMS *m*/*z*: 597.1443 [M + H]^+^; ^1^H-NMR (600 MHz, DMSO-*d*_6_): 7.29 (1H, s, H-6′), 7.07 (1H, s, H-3), 6.74 (1H, s, H-3′), 6.72 (1H, s, H-8), 6.43 (1H, s, H-6), 5.08 (1H, d, *J* = 7.2 Hz, H-1′′), 4.88 (1H, d, *J* = 6.6 Hz, H-1′′′); ^13^C-NMR (150 MHz, DMSO-*d*_6_): 181.9 (C-4), 162.7 (C-7), 161.4 (C-2), 160.9 (C-5), 156.9 (C-9), 150.1 (C-4′), 149.8 (C-2′), 140.4 (C-5′), 114.3 (C-6′), 110.4 (C-1′), 108.5 (C-3), 105.0 (C-3′), 104.1 (C-10), 101.9 (C-1′′′), 99.6 (C-1′′), 99.1 (C-6), 94.3 (C-8), 76.9 (C-5′′′), 76.2 (C-3′′), 76.1 (C-3′′′), 73.0 (C-2′′′), 72.9 (C-2′′), 69.3 (C-4′′′), 69.1 (C-4′′), 65.6 (C-5′′), 60.4 (C-6′′′).

*Isoetin-7-glucoside-2′-arabinoside* (**6**): C_26_H_28_O_16_; ESI/LTQ-Orbitrap-HRMS *m*/*z*: 597.1448 [M + H]^+^; ^1^H-NMR (600 MHz, DMSO-*d*_6_): 7.30 (1H, s, H-6′), 7.16 (1H, s, H-3), 6.75 (1H, s, H-3′), 6.72 (1H, s, H-8), 6.42 (1H, d, *J* = 1.8 Hz, H-6), 5.09 (1H, d, *J* = 7.2 Hz, H-1′′), 4.86 (1H, d, *J* = 6.6 Hz, H-1′′′); ^13^C-NMR (150 MHz, DMSO-*d*_6_): 182.2 (C-4), 162.9 (C-7), 161.6 (C-2), 161.2 (C-5), 157.1 (C-9), 150.2 (C-4′, 2′), 140.5 (C-5′), 114.6 (C-6′), 110.4 (C-1′), 108.8 (C-3), 105.2 (C-3′), 104.2 (C-10), 101.6 (C-1′′′), 99.8 (C-1′′), 99.3 (C-6), 94.5 (C-8), 77.1 (C-5′′′), 76.4 (C-3′′′), 73.1 (C-2′′), 72.3 (C-2′′′), 70.5 (C-3′′), 69.5 (C-4′′′), 67.1 (C-4′′), 65.2 (C-5′′), 60.6 (C-6′′′).

*Caffeic acid* (**7**): C_9_H_8_O_4_; ESI/LTQ-Orbitrap-HRMS *m*/*z*: 179.0340 [M + H]^+^; ^1^H-NMR (600 MHz, DMSO-*d*_6_): 7.43 (1H, m, H-7), 7.02 (1H, s, H-2), 6.96 (1H, m, H-6), 6.76 (1H, d, *J* = 7.8 Hz, H-5), 6.19 (1H, m, H-8); ^13^C-NMR (150 MHz, DMSO-*d*_6_): 168.5 (C-9), 148.6 (C-4), 146.1 (C-3), 145.1 (C-7), 126.2 (C-1), 121.7 (C-6), 116.3 (C-5), 115.6 (C-2), 115.1 (C-8).

*1-caffeoylglycerol* (**8**): C_12_H_14_O_6_; ESI/LTQ-Orbitrap-HRMS *m*/*z*: 253.0340 [M − H]^−^; ^1^H-NMR (600 MHz, DMSO-*d*_6_): 7.49 (1H, t, *J* = 7.8 Hz, H-7), 7.05 (1H, s, H-2), 6.99 (1H, d, *J* = 7.8 Hz, H-6), 6.76 (1H, d, *J* = 7.2 Hz, H-5), 6.26 (1H, t, *J* = 12.6, Hz, H-8), 4.15 (1H, m, H-1′a), 4.01 (1H, m, H-1′b), 3.70 (1H, m, H-2′), 3.38 (1H, m, H-3′); ^13^C-NMR (150 MHz, DMSO-*d*_6_): 166.8 (C-9), 148.7 (C-7), 145.8 (C-4), 145.3 (C-3), 125.6 (C-1), 121.6 (C-6), 116.0 (C-5), 114.9 (C-2), 114.1 (C-8), 69.6 (C-2′), 65.8 (C-1′), 62.9 (C-3′).

*Ferulic acid* (**9**): C_10_H_10_O_4_; ESI/LTQ-Orbitrap-HRMS *m*/*z*: 193.0497 [M − H]^−^; ^1^H-NMR (600 MHz, DMSO-*d*_6_): 7.46 (1H, m, H-7), 7.24 (1H, s, H-2), 7.05 (1H, d, *J* = 8.4 Hz, H-6), 6.75 (1H, d, *J* = 8.4 Hz, H-5), 6.33 (1H, d, *J* = 15.6 Hz, H-8), 3.77 (1H, s, H-10); ^13^C-NMR (150 MHz, DMSO-*d*_6_): 168.0 (C-9), 149.1 (C-4), 147.9 (C-3), 144.5 (C-7), 125.8 (C-1), 122.8 (C-6), 115.6 (C-5), 115.5 (C-8), 111.1 (C-2), 55.7 (C-10).

*Chlorogenic acid* (**10**): C_12_H_14_O_6_; ESI/LTQ-Orbitrap-HRMS *m*/*z*: 353.0875 [M − H]^−^; ^1^H-NMR (600 MHz, DMSO-*d*_6_): 7.41 (1H, d, *J* = 15.6 Hz, H-7′), 7.02 (1H, m, H-2′), 6.98 (1H, m, H-6′), 6.76 (1H, d, *J* = 8.4 Hz, H-5′), 6.15 (1H, d, *J* = 15.6 Hz, H-8′), 5.07 (1H, d, *J* = 5.7 Hz, H-3), 3.91 (1H, s, H-5), 3.55 (1H, s, H-4), 2.02 (2H, m, H-2), 2.02(1H, m, H-6a), 1.78 (1H, m, H-6b); ^13^C-NMR (150 MHz, DMSO-*d*_6_): 175.46 (C-7), 166.28 (C-9′), 148.86 (C-4′), 146.07 (C-3′), 145.48 (C-7′), 126.10 (C-1′), 121.93 (C-6′), 116.25 (C-5′), 115.21 (C-2′), 114.79 (C-8′), 73.99 (C-1), 71.39 (C-5), 70.84 (C-4), 68.59 (C-3), 37.69 (C-2), 36.75 (C-6).

*Chicoric acid* (**11**): C_22_H_18_O_12_; ESI/LTQ-Orbitrap-HRMS *m*/*z*: 473.0719 [M − H]^−^; ^1^H-NMR (600 MHz, MeOH-d4): 7.47 (2H, d, *J* = 11.4 Hz, H-7′, 7′′), 7.05 (2H, s, H-2′, 2′′), 6.94 (2H, s, H-6′, 6′′), 6.75 (2H, s, H-5′, 5′′), 6.27 (2H, d, *J* = 15.0 Hz, H-8′, 8′′), 5.54 (2H, s, H-2, 3); ^13^C-NMR (150 MHz, DMSO-*d*_6_): 172.4 (C-1, 4), 166.7 (C-9′, 9′′), 147.5 (C-4′, 4′′), 145.2 (C-3′, 3′′), 144.7 (C-7′, 7′′), 125.7 (C-1′, 1′′), 121.0 (C-6′, 6′′), 144.5 (C-5′, 5′′), 113.3 (C-2′, 2′′), 113.1 (C-8′, 8′′), 73.6 (C-2, 3).

### 3.5. UHPLC-ESI/LTQ-Orbitrap-HRMS Conditions

A UHPLC-ESI/LTQ-Orbitrap-HRMS was used to determine the molecular weights of the isolated compounds. The samples were dissolved in distilled water. The column (Hypersil GOLD C18, 2.1 × 50 mm, 1.9 μm, Thermo) and sampler temperatures were maintained at 30 and 15 °C, respectively. Ultraviolet (UV) radiation was not used. 

Solvent A (0.1% formic acid in water) and solvent B (0.1% formic acid in acetonitrile) were used as gradient linear mobile phases (0–18 min, 0–50% B; 18–20 min, 50–100% B). All eluents were filtered with a 0.45 μm polyvinylidene fluoride (PVDF) syringe filter. The flow rate was adjusted to 0.3 mL/min. The injection volume was 5.0 µL for the standard solution that was used. The optimized conditions for the analysis were as follows: heater temperature, 300 °C; capillary temperature, 360 °C; aux gas flow rate, 10 L/h; sheath gas flow rate, 45 L/h; S-lens RF level, 50.0 V; spray capillary voltage, 3.0 kV; full MS resolution, 35,000 (FWHM @ *m*/*z* 200); full MS AGC target, 3e^6^; and full MS maximum IT, 200 ms.

### 3.6. Measurement of BSA-glucose and Fructose Inhibitory Activity

Inhibition of AGE formation in BSA-glucose system was determined using the spectrophotometric method described previously [37]. All test samples were dissolved in 10% DMSO. The assay mixture was 50 mM phosphate buffer (pH 7.4) with 0.02% sodium azide, BSA (10 mg/mL), 0.4 M d-fructose and d-glucose, and sample or buffer. This mixture was incubated at 60 °C for 2 days. After incubation, the fluorescence was measured (excitation and emission wavelengths 350 and 450 nm, respectively) in a 96-black well plate. We used AG as a positive control. Three replicate samples were run for each set. The inhibitory activity on AGE formation was calculated using the following formula: {(Ac − As)/Ac} × 100, where Ac is fluorescence of the control, and As is the fluorescence of the sample. 

### 3.7. Measurement of BSA-MGO Inhibitory Activity

This assay was performed according to a previously described method, with modifications [38]. The inhibitory effect on protein glycation induced by MGO (40% aqueous solution) and the main reactive intermediate compound formed in Maillard reaction were evaluated. All test samples were dissolved in 10% DMSO. The assay mixture was 50 mM phosphate buffer (pH 7.4) with 0.02% sodium azide, BSA (1 mg/mL), 7 mM MGO, and sample or buffer. This mixture was incubated at 60 °C for 2 days. After incubation, fluorescence was measured on (excitation and emission wavelengths of 340 and 420 nm, respectively) in a 96-black well plate. We used AG as a positive control. Three replicate samples were run for each set. The inhibitory activity was calculated using the same equation applied in BSA-glucose assay.

### 3.8. Measurement α-Glucosidase Inhibitory Activity

This assay was performed using a 96-well microplate reader and a spectrophotometer. The reaction mixture contained 100 mM phosphate buffer (pH 6.8), 2.5 mM p-NPG, and sample or buffer. A chromogenic substance was used for quantification purposes. After the addition of mixture solution to each well, 10 mM phosphate buffer (20 µL, pH 6.8) containing 0.2 U/mL α-glucosidase was added. This mixture was incubated at 37 °C for 15 min. The reaction was stopped by adding 80 µL of 0.2 mol/L sodium carbonate solution. The absorbance was measured at 405 nm immediately after stopping the reaction using a SUNRISE microplate reader. Acarbose dissolved in 10% DMSO was used as the positive control. Three replicate samples were run for each set. The inhibitory activity was calculated using the equation applied in BSA-glucose assay, but absorbance was measured instead of fluorescence.

### 3.9. Statistical Analysis

All assays were performed in triplicate. Data were presented as mean ± standard deviation (SD) and analyzed by using one-way ANOVA. The data were considered to have statistical significance at *p* < 0.05.

### 3.10. HPLC Analysis

To identify the six major compounds from *T. coreanum,* a Waters Kromasil column C18 (4.6 × 250 mm, 5 μm) column was used for determination major compounds in *T. coreanum*. Solvent A (0.2% phosphoric acid in water) and solvent B (methanol) were used as gradient linear mobile phases (0–15 min, 93–81% B; 15–25 min, 81–60% B; 25–45 min, 60–40% B) at a mobile phase rate of 1 mL/min. All eluents were filtered with a 0.45 μm PVDF syringe filter. The injection volume was 10 μL, and compounds were measured at a wavelength of 330 nm. For preparation of extract stock solutions, plant powders were sonicated with MeOH for 60 min and dried under vacuum by using a rotary evaporator at 50 °C. Then, they were dissolved in MeOH to a concentration of 10,000 ppm. Standard compound stock solutions were dissolved in MeOH. All analyzed solutions were strained using a 0.45 μm PVDF membrane filter prior to injection. The standard calibration curve was constructed using five concentrations. The linear relationship between peak area and concentration is presented in Table 6. The concentrations of the six major compounds were calculated using regression equations based on the calibration curves. To optimize the extraction conditions, a patent extraction method was performed using MeOH and EtOH with different solvent compositions (30%, 50%, 70%, and 100%) and extracted for 30, 60, 90, and 120 min, respectively. 

## 4. Conclusions

After confirming that EA, BuOH, and water fraction of *T. coreanum* exhibited the strongest inhibitory capacities, we isolated the 11 major bioactive compounds of *T. coreanum* and characterized their structures. The isolated bioactive compounds inhibited AGE formation in two systems and α-glucosidase activity, which is related to diabetes and its complications. Compounds **3**, **4**, and **11** exhibited stronger inhibitory activity than other compounds against α-glucosidase. All compounds except compound **9** showed a stronger inhibition than the positive control and compound **11** showed the strongest activity in the BSA-glucose system. In the BSA-MGO system, all compounds showed a higher inhibition rate than the positive control. Compound **11** exhibited higher AGE-inhibitory activity than the positive control in the two systems and showed higher inhibitory potency against α-glucosidase, followed by compound **10**. Compounds **1**, **2**, **7**, **9**–**11** with a high inhibitory effect were investigated content of *T. coreanum* from variety region in Korea and *T. officinale* using HPLC. *T. coreanum* showed higher content of these bioactive compounds than *T. officinale*. Indeed, *T. coreanum* showed stronger inhibition of AGE formation and α-glucosidase activity than *T. officinale*. We suggested that *T. coreanum* was superior to *T. officinale* and that compounds **10** and **11** were the major components, which are responsible for the antidiabetic effects of *T. coreanum*. We also suggested that 30–90 min extraction with 50% ethanol was the optimized solvent condition and *T. coreanum* harvested from Gyeongsangnam-do, Sancheong was the most useful natural alternative medicine for diabetic complications. Based on these results, we demonstrated the preventive and therapeutic efficacy of *T. coreanum* and its potential use as a cost-effective phytopharmaceutical medicine in complementary therapy against type-2 diabetes and its complications. It is required to evaluate whether further research, including in vivo studies and clinical trials on the efficacy of these isolated compounds, are sufficient to the compounds be used in clinical applications. In addition, the isolated compound may have a beneficial therapeutic effect on other diseases.

## Figures and Tables

**Table 1:** IC_50_ of the extracts and fractions from *T. coreanum* on advanced glycation end-product (AGE) formation in BSA/glucose and BSA/ MGO systems and α-glucosidase inhibitory activities.

Sample	IC_50_ *^a^* (μg/mL)		
	BSA/Glucose	BSA/MGO	α-Glucosidase
Ext.	360.41 ± 23.58 ***	311.33 ± 14.86 ***	1623.08 ± 184.40 ***
Hx fr.	ND *^d^*	529.24 ± 4.75 *	1270.70 ± 72.58 ***
DCM fr.	297.10 ± 5.26 **	247.47 ± 23.87 ***	2041.44 ± 469.46 **
EA fr.	119.47 ± 12.06 **	127.31 ± 5.51 ***	554.53 ± 36.46 ***
BuOH fr.	333.71 ± 27.02 **	171.40 ± 69.28 *	ND *^d^*
Water fr.	435.60 ± 59.57 **	213.96 ± 3.38 ***	ND *^d^*
*T. officinale*	201.25 ± 33.99 ***	105.37 ± 24.42 **	2195.89 ± 267.35 **
*T. coreanum*	183.97 ± 28.52 **	98.82 ± 2.51 *	444.97 ± 55.86 **
AMG *^b^*	218.57 ± 24.38 ***	67.51 ± 5.27 **	-
Acarbose *^c^*	-	-	116.86 ± 7.23 **

Data are presented as mean ± SD (*n* = 3). *^a^* IC_50_ calculated from the least-squares regression line of the logarithmic concentrations plotted against the residual activity. *^b^* AMG was used as a positive control of AGE formation inhibitory activities. *^c^* Acarbose was used as a positive control of α-glucosidase inhibitory activity. *^d^* ND: not detected. * Indicates a significant difference from control; * *p* < 0.5, ** *p* < 0.05, *** *p* < 0.005. -: not measured.

**Table 2:** Identification of compounds **1**–**11** in *T. coreanum* by UHPLC-ESI/LTQ-Orbitrap-HRMS analysis.

Compound No.	Rt (min)	Formula	Mass Mode	Theoretical Mass	Observed Mass	Ass Error (Da)	Mass Accuracy (ppm)
**1**	6.86	C_15_H_10_O_6_	Positive	287.0550	287.0546	0.0004	1.4
**2**	5.89	C_21_H_20_O_11_	Positive	449.1078	449.1076	0.0002	0.4
**3**	6.36	C_21_H_20_O_11_	Positive	449.1078	449.1076	0.0002	0.4
**4**	5.86	C_27_H_30_O_15_	Positive	595.1657	595.1650	0.0007	1.2
**5**	5.01	C_26_H_28_O_16_	Positive	597.1450	597.1448	0.0002	0.3
**6**	5.26	C_26_H_28_O_16_	Positive	597.1450	597.1443	0.0007	1.2
**7**	5.01	C_26_H_28_O_16_	Positive	597.1450	597.1448	0.0002	0.3
**8**	4.30	C_9_H_8_O_4_	Negative	179.0339	179.0340	0.0001	0.6
**9**	5.78	C_12_H_14_O_6_	Negative	193.0495	193.0497	0.0002	1.0
**10**	4.87	C_10_H_10_O_4_	Negative	253.0707	253.0714	0.0007	1.4
**11**	4.13	C_16_H_18_O_9_	Negative	353.0867	353.0875	0.0008	2.3

**Table 3:** IC_50_ of the compounds **1**–**11** from *T. coreanum* on advanced glycation end-product (AGE) formation and α-glucosidase inhibitory activities.

Compound	IC_50_ *^a^* (μM)		
	BSA/Glucose	BSA/MGO	α-Glucosidase
**1**	236.48 ± 9.11 **	66.11 ± 17.06 **	ND
**2**	122.81 ± 2.02 ***	107.83 ± 8.14 **	1455.95 ± 126.32 ***
**3**	423.30 ± 18.04 **	90.81 ± 21.74 **	598.24 ± 146.52 *
**4**	253.31 ± 5.10 **	135.65 ± 2.64 **	670.50 ± 50.83 ***
**5**	268.18 ± 3.41 **	129.79 ± 28.27 **	ND *^d^*
**6**	238.05 ± 13.82 ***	148.37 ± 36.29 **	ND *^d^*
**7**	324.21 ± 8.29 **	79.65 ± 23.45 ***	5134.55 ± 803.54 **
**8**	306.99 ± 10.16 **	151.67 ± 65.36 **	2951.13 ± 3.94 *
**9**	704.86 ± 167.43 *	140.72 ± 67.78 **	ND *^d^*
**10**	83.62 ± 55.49 ***	138.18 ± 1.91 **	1148.67 ± 162.05 **
**11**	64.70 ± 16.73 **	141.21 ± 8.76 **	639.25 ± 12.51 ***
AMG *^b^*	601.53 ± 50.35 ***	295.21 ± 42.67 ***	-
Acarbose *^c^*	-	-	355.86 ± 17.25 ***

Data are presented as mean ± SD (*n* = 3). *^a^* IC_50_ calculated from the least-squares regression line of the logarithmic concentrations plotted against the residual activity. *^b^* AMG was used as a positive control of AGE formation inhibitory activity. *^c^* Acarbose was used as a positive control of α-glucosidase inhibitory activity. *^d^* ND: not detected. * Indicates a significant difference from control; * *p* < 0.5, ** *p* < 0.05, *** *p* < 0.005. -: not measured.

**Table 4:** Content of compounds **1**, **2**, **7**, **9**–**11** with respect to different solvent compositions and extraction time. MeOH: methanol; EtOH: ethanol.

Solvent Composition	Compound 1 (mg/g)	Compound 2 (mg/g)	Compound 7 (mg/g)	Compound 9 (mg/g)	Compound 10 (mg/g)	Compound 11 (mg/g)
30% MeOH 60 min	0.31 ± 0.07	0.49 ± 0.04	0.47 ± 0.13	4.58 ± 0.77	3.64 ± 0.35	131.98 ± 9.02
50% MeOH 60 min	0.17 ± 0.05	0.65 ± 0.09	0.36 ± 0.09	4.66 ± 0.45	4.81 ± 0.45	181.58 ± 9.80
70% MeOH 60 min	0.21 ± 0.03	0.61 ± 0.11	0.46 ± 0.04	4.71 ± 0.35	4.66 ± 0.72	173.37 ± 11.15
100% MeOH 60 min	0.20 ± 0.02	0.32 ± 0.02	0.23 ± 0.02	4.53 ± 0.10	1.59 ± 0.19	46.06 ± 4.44
30% EtOH 60 min	0.27 ± 0.04	0.73 ± 0.06	0.60 ± 0.06	4.62 ± 0.37	4.35 ± 0.31	161.46 ± 5.19
50% EtOH 60 min	0.39 ± 0.09	0.76 ± 0.07	0.47 ± 0.06	4.69 ± 0.45	5.40 ± 0.19	204.98 ± 12.60
70% EtOH 60 min	0.20 ± 0.07	0.68 ± 0.09	0.46 ± 0.01	4.68 ± 0.75	5.23 ± 0.26	203.60 ± 0.98
100% EtOH 60 min	0.16 ± 0.04	0.30 ± 0.04	0.35 ± 0.05	4.39 ± 0.65	0.90 ± 0.33	33.65 ± 4.45
70% MeOH 30 min	0.19 ± 0.05	0.61 ± 0.04	0.50 ± 0.04	4.77 ± 0.41	5.15 ± 0.44	206.12 ± 3.14
70% MeOH 60 min	0.41 ± 0.10	0.79 ± 0.09	0.49 ± 0.09	4.75 ± 0.43	5.19 ± 0.49	210.91 ± 2.24
70% MeOH 90 min	0.24 ± 0.01	0.75 ± 0.11	0.48 ± 0.12	4.76 ± 0.39	4.99 ± 0.30	208.72 ± 1.15
70% MeOH 120 min	0.27 ± 0.03	0.73 ± 0.21	0.60 ± 0.14	4.62 ± 0.45	4.35 ± 0.41	161.46 ± 0.95

Data are mean ± SD (*n* = 3) in mg/g dried sample.

**Table 5:** Content of compounds **1**, **2**, **7**, **9**, **10** by region in Korea.

Sample	Contents (mg/g)					
	1	2	7	9	10	11
Gyeongsangnam-do, Sancheong	83.33 ± 0.14	28.30 ± 0.09	4.08 ± 0.09	4.59 ± 0.15	12.82 ± 0.21	562.36 ± 6.21
Gyeongsangbuk-do, Yeongcheon	41.37 ± 0.09	44.10 ± 0.11	1.23 ± 0.05	4.45 ± 0.09	20.32 ± 0.66	215.27 ± 0.2.11
Gyeonggi-do, Gimpo	5.08 ± 0.04	6.40 ± 0.04	1.17 ± 0.01	4.39 ± 0.11	2.02 ± 0.04	46.33 ± 0.96
Chungcheongnam-do, Taean	7.05 ± 0.02	15.87 ± 0.12	5.19 ± 0.05	4.47 ± 0.19	2.75 ± 0.04	106.10 ± 2.64
Jeollabuk-do, Jeongeup	7.10 ± 0.05	13.24 ± 0.07	3.69 ± 0.01	4.62 ± 0.16	10.53 ± 0.02	230.07 ± 2.98
Gangwon-do, Yanggu	1.23 ± 0.02	1.38 ± 0.09	1.52 ± 0.01	4.48 ± 0.05	4.54 ± 0.04	70.16 ± 1.65
Gangwon-do, Yanggu	1.06 ± 0.01	2.03 ± 0.09	2.80 ± 0.03	4.54 ± 0.04	24.11 ± 0.47	230.27 ± 3.11
Gyeongsangbuk-do, Bonghwa	10.10 ± 0.08	17.66 ± 0.09	2.57 ± 0.09	4.56 ± 0.04	13.14 ± 0.69	180.62 ± 2.64

Data are mean ± SD (*n* = 3) in mg/g dried sample.

**Table 6:** Linear relation between peak area and concentration (*n* = 3).

Compound Number	Rt (min)	Regression Equation	*r* ^2^	Linear Range (µg/mL)	LOD (µg/mL)	LOQ (µg/mL)
**1**		y=24,645x+3862.8	0.9999	0.5–50	0.12	0.36
**2**		y=11,593x+2829.2	0.9999	0.5–50	0.24	0.73
**7**		y=42,466x−819.39	0.9999	0.5–50	0.22	0.67
**9**		$y=442,002x-\left(2\times 10^{6}\right)$	0.9728	0.5–50	0.27	0.82
**10**		y=26,896x−4511.4	0.9998	0.5–50	0.13	0.40
**11**		y=16,122x−2382.2	0.9999	0.5–50	0.31	0.95
